# Discovery of Phloeophagus Beetles as a Source of *Pseudomonas* Strains That Produce Potentially New Bioactive Substances and Description of *Pseudomonas bohemica* sp. nov.

**DOI:** 10.3389/fmicb.2018.00913

**Published:** 2018-05-08

**Authors:** Zaki Saati-Santamaría, Rubén López-Mondéjar, Alejandro Jiménez-Gómez, Alexandra Díez-Méndez, Tomáš Větrovský, José M. Igual, Encarna Velázquez, Miroslav Kolarik, Raúl Rivas, Paula García-Fraile

**Affiliations:** ^1^Microbiology and Genetics Department, University of Salamanca, Salamanca, Spain; ^2^Spanish-Portuguese Institute for Agricultural Research (CIALE), Salamanca, Spain; ^3^Institute of Microbiology of the Czech Academy of Sciences, Vestec, Czechia; ^4^Institute of Natural Resources and Agrobiology of Salamanca, IRNASA-CSIC, Salamanca, Spain; ^5^Associated R&D Unit, USAL-CSIC (IRNASA), Salamanca, Spain

**Keywords:** antimicrobials, anticarcinogenic, antiviral, genome mining, bark beetles, antibiotic resistance, NRPS-PKS, secondary metabolites

## Abstract

Antimicrobial resistance is a worldwide problem that threatens the effectiveness of treatments for microbial infection. Consequently, it is essential to study unexplored niches that can serve for the isolation of new microbial strains able to produce antimicrobial compounds to develop new drugs. Bark beetles live in phloem of host trees and establish symbioses with microorganisms that provide them with nutrients. In addition, some of their associated bacteria play a role in the beetle protection by producing substances that inhibit antagonists. In this study the capacity of several bacterial strains, isolated from the bark beetles *Ips acuminatus, Pityophthorus pityographus Cryphalus piceae*, and *Pityogenes bidentatus*, to produce antimicrobial compounds was analyzed. Several isolates exhibited the capacity to inhibit Gram-positive and Gram-negative bacteria, as well as fungi. The genome sequence analysis of three *Pseudomonas* isolates predicted the presence of several gene clusters implicated in the production of already described antimicrobials and moreover, the low similarity of some of these clusters with those previously described, suggests that they encode new undescribed substances, which may be useful for developing new antimicrobial agents. Moreover, these bacteria appear to have genetic machinery for producing antitumoral and antiviral substances. Finally, the strain IA19^T^ showed to represent a new species of the genus *Pseudomonas*. The 16S rRNA gene sequence analysis showed that its most closely related species include *Pseudomonas lutea, Pseudomonas graminis, Pseudomonas abietaniphila* and *Pseudomonas alkylphenolica, with* 98.6, 98.5 98.4, and 98.4% identity, respectively. MLSA of the housekeeping genes *gyr*B, *rpo*B, and *rpo*D confirmed that strain IA19^T^ clearly separates from its closest related species. Average nucleotide identity between strains IA19^T^ and *P. abietaniphila* ATCC 700689^T^, *P. graminis* DSM 11363^T^, *P. alkylphenolica* KL28^T^ and *P. lutea* DSM 17257^T^ were 85.3, 80.2, 79.0, and 72.1%, respectively. Growth occurs at 4-37°C and pH 6.5-8. Optimal growth occurs at 28°C, pH 7–8 and up to 2.5% NaCl. Respiratory ubiquinones are Q9 (97%) and Q8 (3%). C16:0 and in summed feature 3 are the main fatty acids. Based on genotypic, phenotypic and chemotaxonomic characteristics, the description of *Pseudomonas bohemica* sp. nov. has been proposed. The type strain is IA19^T^ (=CECT 9403^T^ = LMG 30182^T^).

## Introduction

During thousands of years, natural molecules obtained from plants, animals or microorganisms have been the main source of drugs to treat human illnesses. However, the field of chemistry has greatly influenced the availability of drugs by using combinatorial chemistry to produce new molecules (Cragg and Newman, 2009; Mishra and Tiwari, 2011; Dias et al., 2012; Harvey et al., 2015). Currently, natural sources are receiving more attention, as the production of new drugs chemically is becoming more restricted, and it is becoming more difficult to solve the problems that arise in association with Public Health (Ravelo and Braun, 2009; Harvey et al., 2015).

Antimicrobial resistance is a global problem that threatens the effectiveness of the treatment of microbial infections. During the last few decades, the number of multidrug resistant microbes is exponentially increasing, and the WHO warns that if this tendency persists, the number of human deaths derived from microbial infections will be higher by 2050 than those resulting from cancer. This means that antimicrobial resistance is one of the major menaces to public health at the present time (Levy and Marshall, 2004; Hoffman et al., 2015; Premanandh et al., 2016).

The complex characteristics of natural molecules could make them more suitable than chemically synthesized compounds for fighting disease, since they are more similar to the endogenous metabolites of an organism. Also, the complexity of natural molecules sometimes impedes their synthesize using chemical methods (Balamurugan et al., 2005; Ravelo and Braun, 2009; Drewry and Macarron, 2010; Harvey et al., 2015). Therefore, the study of unexplored ecological niches that can serve as a novel source for the isolation of new microbial strains able to produce antimicrobial compounds, which could serve as a basis for the development of new drugs effective in the treatment of microbial infections, is of utmost importance (Kennedy et al., 2007; Cragg and Newman, 2009; Piel, 2011; Harvey et al., 2015).

Bark beetles (*Curculionidae, Scolytinae*) belong to a group of insects that live in and feed on the phloem, the inner layer of the bark, in their host trees (Six, 2012). As many other bark beetles, they establish a symbiotic relationship with microorganisms that provide nutrients to the insect (García-Fraile, 2018). In addition, it has also been reported that some of the bacteria associated with these beetles play a role in the protection of the beetle holobiont –the insect and its microbial symbionts– by producing substances that inhibit the development of pathogens and other antagonists (García-Fraile, 2018).

*Pseudomonas* is a genus of bacteria found in a wide range of habitats (for a revision see Peix et al., 2018) and also includes bacteria that are commonly associated with bark beetles (Adams et al., 2009; Boone et al., 2013; Hu et al., 2014; Menéndez et al., 2015; Xu et al., 2015, 2016). Many strains belonging to this genus are used as biocontrol agents for inhibiting some plant pathogens (for a revision see Olorunleke et al., 2015), which further supports their capability to produce useful antimicrobials. Moreover, many drugs coming from *Pseudomonas* strains with interesting clinical applications have been described. Thus, the search for *Pseudomonas* strains associated with bark beetles is interesting from the point of view of discovering new antimicrobials and other compounds that may be of potential use to the pharmaceutical industry.

As regards, the aim of this work was to study the potential of bacterial bark beetles associates to inhibit Gram positive and negative bacteria and fungi. In addition, a genome sequence analysis was carried out to test the capacity of some of the most promising strains belonging to the genus *Pseudomonas* to produce antimicrobial compounds, which could lead to the development of new antibiotics. Finally, based on phenotypic, genotypic and chemotaxonomic tests, we describe one of the new isolates, strain IA19^T^, as a new species of the genus *Pseudomonas*.

## Materials and methods

### Strains used in this study

Bacterial strains used in this study were isolated from adult bark beetles of several species -*Ips acuminatus, Cryphalus piceae, Pityophthorus pityographus*, and *Pityogenes bidentatus* (all Coleoptera, *Scolytinae*)-. The isolation of the bacterial associates of *C. piceae* and *P. pityographus* has been previously described in Fabryová et al. (2018). The newly obtained bacteria isolated in this study were obtained from *I. acuminatus* and *P. bidentatus* beetles, extracted from branches from *Pinus sylvestris* exhibiting the typical boring holes of bark beetles which were collected in May 2016 in Stará Boleslav, Czech Republic (coordinates: 50°12′59.5″N, 14°41′58.4″E). The branches were taken to the laboratory and then, under aseptic conditions, the bark was removed, and the bark beetles were sorted into 5 groups of 3 individuals each. The beetles were crushed using sterile toothpicks in 500 μl of sterile water. Serial dilutions were made using the suspensions obtained, and 100 μl of each dilution were plated onto Nutrient Agar (NA) and Tryptose Soy Agar (TSA).

The plates were incubated at 28°C for 2 weeks, and the emerging bacterial colonies with different morphologies were regularly passed to new plates in order to obtain pure cultures. The isolated strains were stored in a sterile 20% glycerol solution at −80°C and sub-cultured regularly in the corresponding isolation medium.

### Bacterial identification and genotypic analysis

To identify the 21 bacterial isolates obtained in this study, total DNA was extracted and the 16S rRNA gene was amplified and sequenced as previously reported (Rivas et al., 2007). Almost complete (~1,400 bp) 16S rRNA sequences were compared with those available in GenBank using the BLASTn program (Altschul et al., 1990) and EzTaxon tool (Kim et al., 2012). The remaining 40 strains used in the antimicrobial screening were identified as detailed by Fabryová et al. (2018).

The amplification and sequencing of the housekeeping genes *gyr*B, *rpo*B, and *rpo*D was performed as described by Menéndez et al. (2015).

The phylogenetic analysis of the 16S rRNA gene sequence and the concatenated sequences of the *gyr*B, *rpo*B, and *rpo*D housekeeping genes of strain IA19^T^ and all the sequences of the closely related species of the genus *Pseudomonas* was done using the MEGA7 software (Kumar et al., 2016), based on the Clustal_W alignment (Thompson et al., 1994; Larkin et al., 2007). The distances were calculated using the Kimura's two-parameter model (Kimura, 1980) and the phylogenetic trees were generated using maximum-likelihood (ML; Rogers et al., 1998) and neighbor-joining (NJ; Saitou and Nei, 1987) analyses.

The average nucleotide identity (ANI) values between the genome sequence of strain IA19^T^ and the genome sequences of the type strains of the closest related species were estimated by using ANI Calculator in the EZBioCloud (http://www.ezbiocloud.net).

The mol % G+C content of DNA was determined from the complete genome sequence.

### Chemotaxonomic analysis

Biomass for the analysis of fatty acid methyl esters (FAME) and respiratory quinones of strain IA19^T^ and its closest related type was harvested after the strains were cultivated for 2 days on TSA medium at 28°C. For FAME analyses, the cells were collected from the plates and placed into sterile plastic tubes and freeze dried. The extraction of fatty acids was carried out as described by Sasser (1990) and analyzed using the Microbial Identification System (MIDI) Sherlock 6.1 together with the library RTSBA6. Quinones extraction and identification were performed at the Identification of Microorganisms Service at the DSMZ, were they are extracted using methanol:hexane (Tindall, 1990a,b), followed by phase separation into hexane, separated into their different classes by thin layer chromatography on silica gel (Macherey-Nagel Art. No. 805 023), using hexane:tert-butylmethylether (9:1 v/v) as solvent. UV absorbing bands corresponding to the different quinone classes are removed from the plate and analyzed by HPLC.

### Bacterial characterization

Gram-staining of strain IA19^T^ was carried out following the protocol described by Doetsch (1981), and motility was checked by phase-contrast microscopy after growing the cells at 22°C for 48 h in NA. The type of flagellation was determined by electron microscopy as previously described by García-Fraile et al. (2015).

Tryptose Soy Broth (TSB) medium supplemented with 0–10% (w/v) NaCl was used to assay salt tolerance in IA19^T^. The same medium, with an adjusted final pH in the range of 4–10, was used for studying the growth capability of the strain at different pHs; in both cases the cultures were incubated at 28°C for up to 1 week. Also, cells were cultured on TSA plates at 4, 8, 22, 28, 37, and 42°C to determine the temperature range for growth. In all cases the presence of growth was checked during 1 week.

For the catalase test, bacterial cells growing in an TSA agar plate were collected and drops of 30% H_2_O_2_ were added over them to detect the formation of bubbles after 5 min, indicating a positive result. The oxidase test was performed following the protocol described by Kovacs (1956).

Finally, the phenotypic characterization of strain IA19^T^ and the type strains of the closest related species was completed using the API20NE and API50CH (bioMerieux) systems.

### Screening for antimicrobial production

The antimicrobial activity screen using the 61 isolates analyzed in this study was carried out on 6 indicator strains: A Gram-negative bacterium, *Klebsiella oxytoca*, a Gram-positive bacterium, *Arthrobacter phenanthrenivorans* two yeast-like fungi, *Candida humilis* and *Pichia fermentans* and two filamentous fungi, *Aspergillus* sp. and *Fusarium* sp.

To analyze the capability of each of the isolates to inhibit the different bacteria and yeast-like indicator strains, cross streak method was used. Each of our isolates was seeded by a single streak in the center of a NA plate. After 5 days of incubation at 28°C, the plates were seeded with the indicator microorganisms by single streaks perpendicular to the central one. After additional 48h incubation, the growth of each indicator strain was analyzed. To study the inhibition of filamentous fungi, our isolates were seeded by a single streak in the center of a NA plate. After 5 days of pre-incubation at 28°C, fungi were inoculated 2 cm far from the central strike by 0.5 cm mycelia discs. After further incubation under suitable conditions for the fungal strains tested (25°C), the diameters of fungal growth in control and sample plates were measured, and the antifungal effect was evaluated.

### Draft genome sequencing and annotation

The genomic DNA for genome sequencing was obtained from bacterial cells of strains IA19^T^, A2-NA12, and A2-NA13 grown on NA plates and collected after 24 h at 28°C, using the ZR Fungal/Bacterial DNA MiniPrep (Zymo Research).

The draft genome sequences of the selected isolates were obtained by shotgun sequencing on an Illumina MiSeq platform via a paired-end run (2 × 251 bp). The sequence data were assembled using Velvet 1.2.10 (Zerbino and Birney, 2008) and a draft genome was obtained. Gene calling and annotation was performed using RAST 2.0 (Rapid Annotation using Subsystem Technology) (Aziz et al., 2008). The SEED-viewer framework (Overbeek et al., 2014) was used for a first mining of genes related to antimicrobial production genes. Moreover, a more specific and detailed analysis of the presence of gene clusters related to antimicrobial substances productions and other secondary metabolites was performed using antiSMASH 3.0 (Weber et al., 2015).

## Results

### Bacterial isolation and identification

The list of bacterial isolates analyzed in this work, as well as their identification based on their partial (≈1,400 bp) 16S rRNA sequence, is presented in Table 1. The list includes the isolates from *I. acuminatus* and *P. bidentatus*, obtained in this study, and the ones from the bark beetles *C. piceae* and *P. pityographus* obtained in a study that tested their capacity to degrade plant cells (Fabryová et al., 2018).

**Table 1:** List of the bacterial strains analyzed in this study and their capability to inhibit reference microbial strains.

					**Inhibition of:**						
**Isolate**	**Origin**	**Beetle**	**Tree**	**Closest related species based on 16S rRNA sequence similarity**	**Similarity (%)**	***K. oxytoca***	***C. humilis***	***P. fermentans***	***A. phenanthrenivorans***	***Aspergillus* sp**.	***Fusarium* sp**.
P3-NA1	Ref	PP	Ps	*Yersinia ruckeri* ATCC 29473	97.4	−	+	−	−	+	−
P3-NA2	Ref	PP	Ps	*Yersinia ruckeri* ATCC 29473	97.5	−	+	−	−	w	−
A1-NA1	Ref	CP	Aal	*Erwinia billingiae* Eb661	99.7	−	+	+	−	+	−
A1-NA3	Ref	CP	Aal	*Pantoea* sp.	−	−	+	−	−	+	−
A1-NA4	Ref	CP	Aal	*Curtobacterium plantarum* CIP 108988	98.3	−	−	+	−	+	−
A2-NA4	Ref	CP	Aal	*Curtobacterium plantarum* CIP 108988	98.3	−	+	−	−	w	−
A2-NA5	Ref	CP	Aal	*Curtobacterium plantarum* CIP 108988	98.3	−	+	−	−	+	−
A2-NA8	Ref	CP	Aal	*Erwinia billingiae* Eb661	99.7	−	−	+	−	+	−
**A2-NA12**	Ref	**CP**	**Aal**	***Pseudomonas arsenicoxydans*** **VC-1**	**99.9**	−	+	+	**w**	**w**	+
**A2-NA13**	Ref	**CP**	**Aal**	***Pseudomonas arsenicoxydans*** **VC-1**	**99.9**	−	+	+	+	+	+
A3-NA1	Ref	CP	Aal	*Yersinia ruckeri* ATCC 29473	97.5	−	−	−	−	+	−
A3-NA4	Ref	CP	Aal	*Yersinia ruckeri* ATCC 29473	97.5	−	+	−	−	+	−
P2-1	Ref	PP	Ps	*Staphylococcus pasteuri* ATCC 51129	99.9	−	−	+	−	+	−
P2-2	Ref	PP	Ps	*Staphylococcus epidermidis* ATCC 14990	100.0	−	−	+	−	+	−
P2-3	Ref	PP	Ps	*Staphylococcus pasteuri* ATCC 51129	99.7	−	−	+	−	+	−
P2-4	Ref	PP	Ps	*Curtobacterium plantarum* CIP 108988	99.7	−	+	−	−	+	−
P2-5	Ref	PP	Ps	*Curtobacterium plantarum* CIP 108988	99.9	−	−	+	−	+	−
P2-6	Ref	PP	Ps	*Curtobacterium plantarum* CIP 108988	99.9	−	−	−	−	w	−
P2-7	Ref	PP	Ps	*Curtobacterium plantarum* CIP 108988	99.7	−	−	+	−	+	−
P3-1	Ref	PP	Ps	*Curtobacterium plantarum* CIP 108988	99.9	−	+	−	−	+	−
P3-2	Ref	PP	Ps	*Curtobacterium plantarum* CIP 108988	99.5	−	+	−	−	+	−
P3-4	Ref	PP	Ps	*Curtobacterium plantarum* CIP 108988	99.9	−	+	−	−	+	−
P4-2	Ref	PP	Ps	*Staphylococcus epidermidis* ATCC 14990	99.9	−	+	w	−	+	−
P4-3	Ref	PP	Ps	*Staphylococcus epidermidis* ATCC 14990	99.9	−	−	+	−	+	−
P4-4	Ref	PP	Ps	*Staphylococcus epidermidis* ATCC 14990	100.0	−	+	−	−	+	−
P5-3	Ref	PP	Ps	*Staphylococcus pasteuri* ATCC 51129	99.9	−	+	−	−	+	−
P5-4	Ref	PP	Ps	*Staphylococcus epidermidis* ATCC 14990	99.9	−	−	+	−	w	−
P5-5	Ref	PP	Ps	*Staphylococcus epidermidis* ATCC 14990	100.0	−	−	+	−	+	−
P5-11	Ref	PP	Ps	*Erwinia billingiae* Eb661	99.7	−	−	−	−	+	−
A1-1	Ref	CP	Aal	*Erwinia billingiae* Eb661	99.6	−	−	−	−	+	−
A1-2	Ref	CP	Aal	*Erwinia billingiae* Eb661	99.4	−	+	−	−	+	−
A1-3	Ref	CP	Aal	*Erwinia billingiae* Eb661	99.6	−	+	−	−	w	−
A2-2	Ref	CP	Aal	*Erwinia billingiae* Eb661	99.7	−	+	−	−	+	−
A2-5	Ref	CP	Aal	*Curtobacterium plantarum* CIP 108988	98.3	−	+	−	−	w	−
A3-2	Ref	CP	Aal	*Yersinia ruckeri* ATCC 29473	97.4	−	−	+	−	+	−
A3-3	Ref	CP	Aal	*Erwinia billingiae* Eb661	99.7	−	−	+	−	+	−
A3-7	Ref	CP	Aal	*Erwinia billingiae* Eb661	99.6	−	+	+	−	+	−
A3-8	Ref	CP	Aal	*Erwinia billingiae* Eb661	99.5	−	+	−	−	+	−
A3-9	Ref	CP	Aal	*Erwinia billingiae* Eb661	99.5	−	+	−	−	+	−
A4-1	Ref	CP	Aal	*Staphylococcus epidermidis* ATCC 14990	99.9	−	−	+	−	+	−
IA2	N	IA	Ps	*Pseudomonas lutea* DSM 17257(T)	98.6	−	+	+	w	w	w
IA3	N	IA	Ps	*Micrococcus yunnanensis* YIM 65004 (T)	99.7	−	+	+	−	+	−
IA4	N	IA	Ps	*Pseudomonas lutea* DSM 17257(T)	98.6	−	+	w	−	+	−
IA5	N	IA	Ps	*Pseudomonas koreensis* AF 468452	99.4	−	+	w	−	+	+
IA6	N	IA	Ps	*Pseudomonas yamanorum* 8H1 (T)	99.9	−	+	+	−	+	+
IA7	N	IA	Ps	*Arthrobacter globiformis* NBRC 12137 (T)	99.5	−	+	+	−	w	−
IA9	N	IA	Ps	*Pseudomonas yamanorum* 8H1 (T)	99.9	−	+	+	−	w	−
IA12	N	IA	Ps	*Pseudomonas gessardii* DSN 17152 (T)	99.9	−	+	+	−	+	+
IA13	N	IA	Ps	*Pseudomonas lutea* DSM 17257(T)	98.6	w	+	+	−	w	−
IA14	N	IA	Ps	*Pseudomonas lutea* DSM 17257(T)	98.6	−	+	+	w	+	−
IA15A	N	IA	Ps	*Pseudomonas lutea* DSM 17257(T)	98.6	−	+	+	w	+	−
IA15B	N	IA	Ps	*Pseudomonas lutea* DSM 17257(T)	98.6	−	+	+	w	+	−
IA16	N	IA	Ps	*Pseudomonas lutea* DSM 17257(T)	98.6	w	+	+	−	w	−
IA17	N	IA	Ps	*Pseudomonas gessardii* DSN 17152 (T)	99.8	−	+	+	−	+	+
IA18	N	IA	Ps	*Pseudomonas lutea* DSM 17257(T)	98.6	−	+	+	w	w	w
**IA19**	**N**	**IA**	**Ps**	***Pseudomonas lutea*** **DSM 17257(T)**	**98.6**	+	+	+	+	+	+
PB2	N	PB	Ps	*Acidovorax radicis* N35 (T)	99.5	−	−	−	−	+	−
PB4	N	PB	Ps	*Pseudomonas agarici* NCPPB 2298 (T)	98.7	−	+	+	−	+	−
PB6	N	PB	Ps	*Erwinia billingiae* CIP 106121 (T)	100.0	−	+	−	−	+	−
PB8	N	PB	Ps	*Pseudomonas cichoriii* ATCC 10857 (T)	99.2	−	+	+	−	+	−
PB9	N	PB	Ps	*Erwinia typography* DSM 22678 (T)	98.2	−	+	−	−	w	−

*Ref, Isolated previously in Fabryová et al. (2018); N, Novel isolates; PP, Pityophthorus pityographus; CP, Cryphalus piceae; IA, Ips acuminatus; PB, Pityogenes bidentatus; Ps, Pinus sylvestris; Aal, Abies alba; +, It does exist inhibition; −, It does not exist inhibition; w, weakly inhibition. Bold values are for those strains selected for genome sequencing*.

### Phylogenetic analyses and average nucleotide identity (ANI) comparison

Analysis of the 16S rRNA gene sequence of strain IA19^T^ suggested that this isolate was a member of the genus *Pseudomonas* but could also belong to a new species within this genus. The most closely related species were *P. lutea* DSM 17257^T^, (98.6% identity), *P. graminis* DSM 11363^T^, (98.5% identity), *P. abietaniphila* ATCC 700689^T^, (98.4% identity) and *P. alkylphenolica* KL28^T^, (98.4% identity).

**Figure 1:**
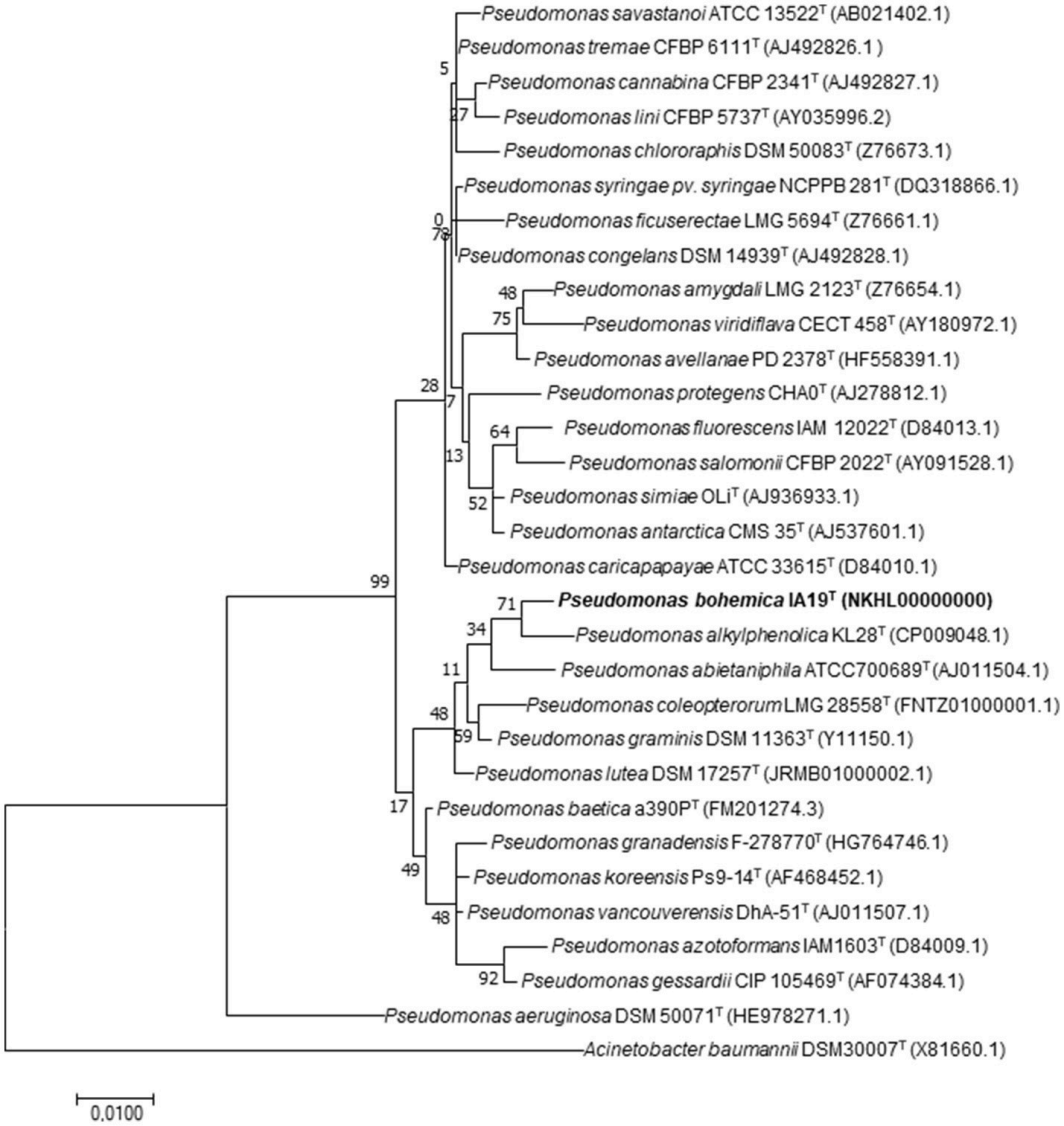
Maximum-likelihood phylogenetic tree based on nearly complete (1,400 bp) 16S rRNA gene sequences of all *Pseudomonas* species closely related to *P. bohemica* IA19^T^ and the species *Acinetobacter baumannii* DSM30007 ^T^, which was included as an outgroup. Bootstrap values (expressed as percentages of 1,000 replications) are shown at the branching points. Scale bar = 1 nucleotide (nt) substitutions per 100 nt. Accession numbers of the sequences are indicated in parentheses.

**Figure 2:**
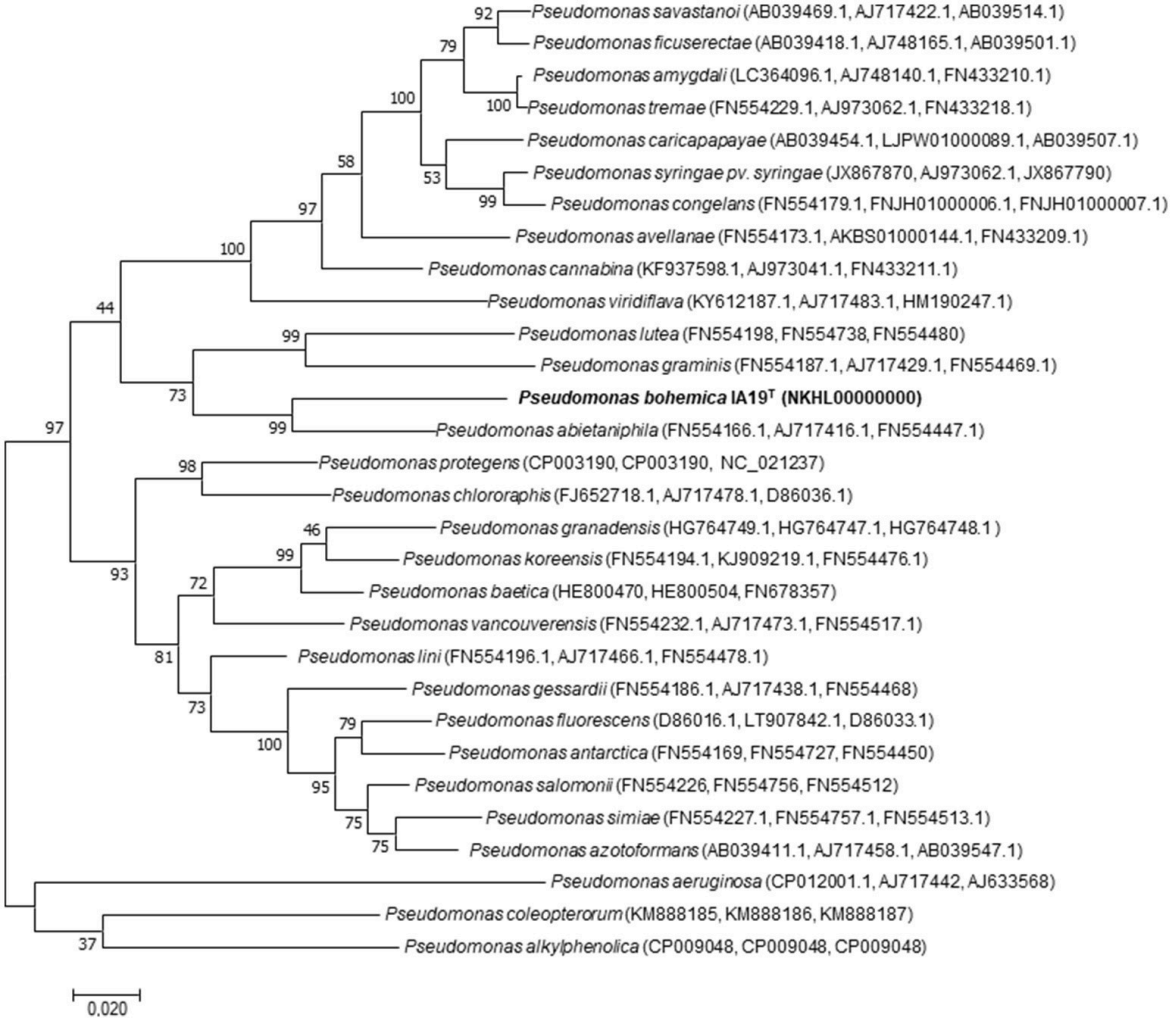
Maximum-likelihood phylogenetic tree based on concatenated partial *gyr*B, *rpo*B, *rpo*D gene sequences of strain *P. bohemica* IA19^T^ and closely related species of the genus *Pseudomonas*. Bootstrap values (expressed as percentages of 1,000 replications) are shown at the branching points. Scale bar = Bar, 2 nucleotide (nt) substitutions per 100 nt. Accession numbers of the sequences are indicated in parentheses.

ML and NJ trees including all related species within the genus *Pseudomonas* showed a similar 16S rRNA sequence phylogenetic clustering, in which strain IA19^T^ clustered with *P. alkylphenolica* KL28^T^ in a broader cluster that also contained *P. lutea* OK2^T^ and *P. graminis* DSM 11363^T^ (Figure 1 and Supplementary Figure 1). Nevertheless, the phylogenetic distances between strain IA19^T^ and its closest related species were broader than those distances among several other different species belonging to this genus, suggesting its classification into a different species. However, the limitations of the analysis based on 16S rRNA gene sequences to discriminate the genus *Pseudomonas* sufficiently at the inter-species level have been described (Ramírez-Bahena et al., 2014). Therefore, the Mutli-Locus Sequence Analysis (MLSA) based on the three housekeeping genes *gyr*B, *rpo*B, and *rpo*D was used for species classification in *Pseudomonas* (Ait Tayeb et al., 2005; Mulet et al., 2009, 2010, 2012; Ramos et al., 2013; Toro et al., 2013; Ramírez-Bahena et al., 2014; Menéndez et al., 2015). Both the NJ and ML analysis of the concatenated housekeeping *gyr*B, *rpo*B and *rpo*D genes sequences (Figure 2 and Supplementary Figure 2) showed that the strain IA19^T^ clustered with *P. abitaniphila* DSM 17554^T^, but the phylogenetic distance between both strains clearly indicated that they belonged to different species. The sequence similarities between strains IA19^T^ and *P. abietaniphila* DSM 17554^T^ housekeeping genes *rpo*D, *rpo*B, and *gyr*B were 89.3, 92.4, and 87.5%, respectively. These values were below those commonly found among different but closely related species of the genus *Pseudomonas* (Ramírez-Bahena et al., 2014).

Finally, authenticity of the novel species was confirmed by ANI comparison between strain IA19^T^ and the genome sequence of the type strains of its closest related strains *P. abietaniphila* ATCC 700689^T^ (GenBank access number: FNCO00000000.1, draft genome), *P. graminis* DSM 11363^T^ (GenBank access number: NZ_FOHW01000000.1, draft genome), *P. alkylphenolica* KL28^T^ (GenBank access number: CP009048.1, complete genome) and *P. lutea* DSM 17257^T^ (GenBank access number: FOEV01000000.1, draft genome) showed values of were 85.31, 80.21, 79.0, and 72.15%, respectively (Table 2). Considering that a threshold value of 95–96% ANI has been established for defining a bacterial species (Richter and Rosselló-Móra, 2009), our results indicated that strain IA19^T^ represented a distinct species of the genus *Pseudomonas*.

**Table 2:** Average nucleotide identity (ANI) comparison between strain IA19^T^ and the genome sequence of its closest relatives.

**ANI**	**1 (%)**	**2 (%)**	**3 (%)**	**4 (%)**	**5 (%)**
1	100				
2	72.15	100			
3	80.21	69.83	100		
4	79.00	72.17	75.76	100	
5	85.31	71.69	79.53	77.39	100

*1, P. bohemica IA19^T^ (NKHL00000000); 2, P. lutea LMG 21974 ^T^ (FOEV01000000.1); 3, P. graminis DSM 11363^T^ (NZ_FOHW01000000.1); 4, P. alkylpenolica KL28^T^ (CP009048.1); 5, P. abietaniphila ATCC 700689^T^ (FNCO00000000.1)*.

### Colony and cellular morphology

**Figure 3:**
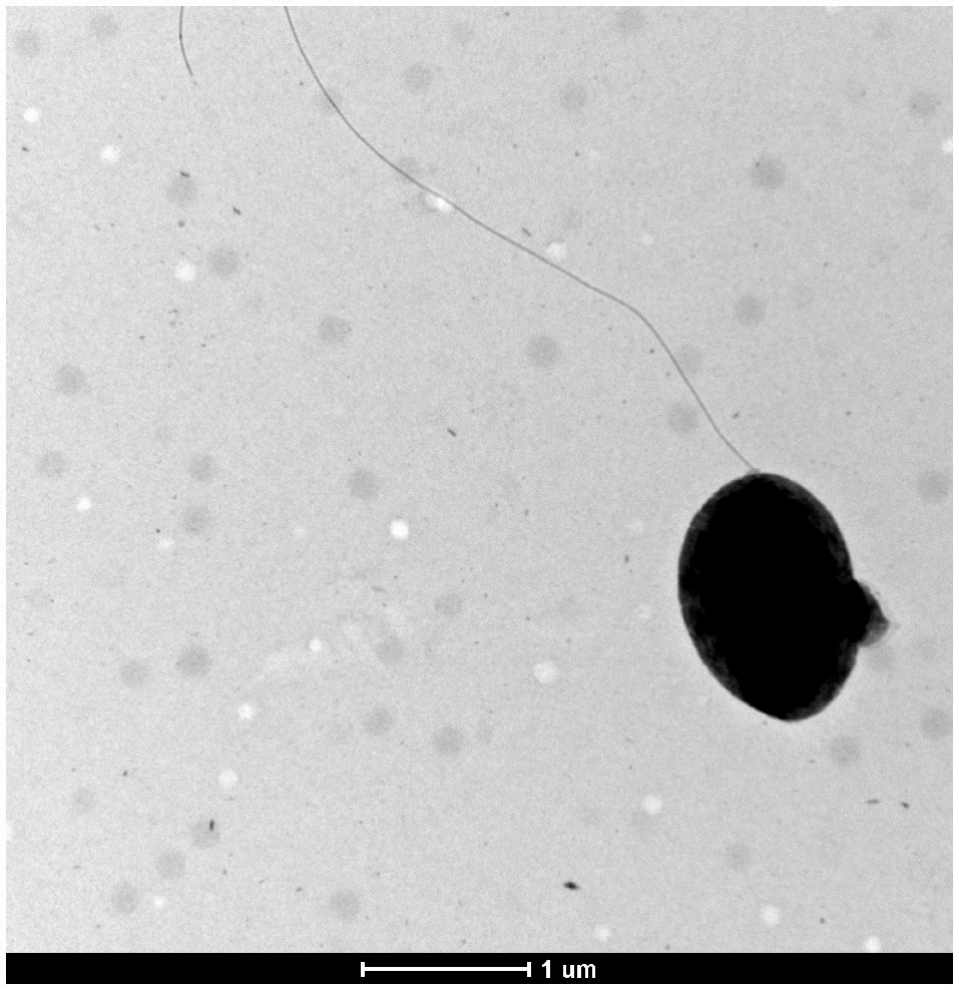
Electron micrograph showing the morphology and flagellation type of *Pseudomonas bohemica* IA19^T^.

The strain IA19^T^ formed round, bright, clear beige and convex colonies with entire border on TSA medium, which were visible after 24 h of incubation at 28°C; these colonies grew to a size of 1–3 mm after 72 h of growth. Also, growth was observed in the temperature range of 4–37°C, where the optimum temperature was at 28°C, and at a pH range of 6.5 and 8, with 7 being the optimum. Cells were Gram-negative, rod-shaped and motile by means of a polar flagellum (Figure 3).

### Phenotypic and chemotaxonomic characterization

The analysis of isopropenoid quinones showed that the isolate IA19^T^ contained ubiquinone-9 (Q9) (97%) as the main respiratory quinone, which is one of the most common quinone systems in those strains belonging to the genus *Pseudomonas* (Oyaizu and Komagata, 1983), and a small amount of ubiquinone-8 (Q8) (3%).

As for its closest related species, the major fatty acids of strain IA19^T^ were 16:0 iso (30.5%) and in summed feature 3 (21.1%), being the whole FAME composition detailed in Table 3.

**Table 3:** Cellular fatty acid composition (%) of *Pseudomonas bohemica* IA19^T^ and its closest related species.

**Fatty acid**	**1**	**2**	**3**	**4**	**5**	**6**
C_10:0_ 3OH	2.6	2.2	1.7	1.5	7.8	3.6
C_12:0_	5.7	5.2	4.6	4.9	3.2	4.8
C_12:0_ 2OH	4.0	3.5	2.9	2.8	7.1	3.7
C_12:0_ 3OH	5.7	4.0	4.1	3.5	7.8	4.5
Sum In Feature 3^¶^	20.1	27.2	38.7	37.2	26.7	20.0
C_16:0_	30.5	24.7	27.7	25.6	27.8	20.5
C_17:0_ cyclo	14.6	10.3	1.3	1.7	6.0	ND
C_17:0_	0.3	1.9	0.3	0.6	ND	TR
Sum In Feature 5^¥^	1.5	0.4	ND	ND	ND	20.0
Sum In Feature 8^§^	11.4	17.3	17.4	21.2	10.5	38.9
C_19:0_ Cyclo w8c	1.3	0.5	ND	ND	ND	ND

*Strains: 1, P. bohemica IA19^T^; 2, P. abietaniphila DSM 17554^T^; 3, P. graminis DSM 11363^T^; 4, P. lutea OK2^T^; 5, P. alkylphenolica KL28^T^ (Mulet et al., 2015); 6, P. aeruginosa ATCC 10145^T^ (Menéndez et al., 2015). Values are percentages of total fatty acids. Those values under 1 per cent for both the strains are not included*.

¶ *Sum in Feature 3: 16:1 w7c/16:1 w6c*.

¥ *Sum in Feature 5: 18:2 w6,9c/18:0 ante*.

§ *Sum in Feature 8: 18:1 w7c/: 18:1 w6c*.

*Values are percentages of total fatty acids*.

*Those values under 1 per cent for both the strains are not included*.

*ND, No Data; TR, Traces*.

The main phenotypic features observed for strain IA19^T^ are detailed in the species description, and the main differences found between this strain and its closest related species, as well as the type species of the genus, *Pseudomonas aeruginosa*, are listed in Table 4.

**Table 4:** Phenotypic differences between *Pseudomonas bohemica* IA19^T^ and its closest related species.

	**1**	**2**	**3**	**4**	**5**	**6**
Arginine dihydrolase	−	+	+	−	+	+
Urease	−	+	+	−	−	d
Hydrolysis of esculin	+	−	+	+	+	−
Asimilation of:						
L-arabinose	+	+	+	+	−	−
Glycerol	+	+	−	+	nd	+
L (+) fructose	+	+	−	nd	+	+
Rhamnose	−	+	−	−	nd	−
Sorbitol	−	+	+	−	nd	−
Sucrose	−	+	+	−	−	−
D-maltose	−	+	−	−	−	−
Capric acid	−	+	+	+	+	nd
Malic acid	−	+	+	+	+	nd
Adonitol	−	+	w	+	−	−
Galactose	−	+	w	+	−	−
Mannose	+	+	−	+	−	−
Meso-inositol	−	+	w	nd	−	−
Manitol	−	+	w	+	−	+
Amygdalin	+	−	−	−	−	nd
Arbutine	+	−	−	−	nd	nd
Salicin	+	−	−	−	nd	nd
Melobiose	−	−	+	+	nd	−
D (-) trehalose	−	+	−	−	−	−
Xylitol	−	+	+	+	−	−
D-Fucose	−	+	+	+	nd	nd
L-Fucose	−	+	+	+	−	−
D-Arabitol	−	+	+	+	−	−
L-Arabitol	−	+	+	+	nd	nd
Gluconate	−	+	+	+	+	−
2-ketogluconate	−	+	+	+	nd	+
5-ketogluconate	−	−	+	−	nd	nd

*Strains: 1, P. bohemica IA19^T^; 2, P. abietaniphila DSM 17554^T^; 3, P. graminis DSM 11363^T^; 4, P. lutea OK2^T^; 5, P. alkylphenolica KL28^T^ (Mulet et al., 2015; Frasson et al., 2017); 6, P. aeruginosa ATCC 10145^T^ (Palleroni, 2005; Clark et al., 2006; Xiao et al., 2009)*.

*+, The bacteria can growth; −, The bacteria cannot growth; w, the bacteria growths weakly; nd, No data; d, variable*.

### *In vitro* detection of the production of antimicrobial substances

From the total of 61 isolates, all showed antimicrobial activity against at least one of the indicator strains (Table 1). With the only exception of two strains, the analyzed isolates from this study inhibited the yeast's growth, 4.9% isolates inhibited the growth of *K. oxytoca* and 13.1% isolates inhibited the growth of *A. phenanthrenivorans*. Moreover, the strain from *Aspergillus* sp. was inhibited by all the strains of this study (Table 1).

Several of the isolates obtained in this study appeared to be potential candidates to produce antimicrobial substances, being several strains identified as *Pseudomonas* some of the best antimicrobial producers, according to our screening. This genus is known to produce antimicrobials and other interesting bioactive compounds (Laine et al., 1996; Stintzi et al., 1996; Raaijmakers et al., 1997; Marinho et al., 2009; Bauer et al., 2015; Nishanth Kumar et al., 2016; Ganne et al., 2017). Based on that, strains *Pseudomonas* IA19^T^, *Pseudomonas* A2-NA12, *Pseudomonas* A2-NA13 were selected for further analysis of their genetic potential to produce pharmaceutically interesting molecules by sequencing and mining their genomes.

### Genomic properties

The size of the genome of the isolate IA19^T^ was estimated to be 6.487 Mb with 5,961 predicted coding sequences, A2-NA12 was estimated to be 5.933 Mb with 5,148 predicted coding sequences and A2- NA13 was estimated to be 5.938 Mb with 5,172 predicted coding sequences. The GC content was predicted to be 59.5 for IA19^T^ and 59.7 for A2-NA12 and A2-NA13. The features of all three genomes are summarized in Table 5.

**Table 5:** Features of the draft genomes of the three *Pseudomonas* isolates sequenced in this study.

**Characteristic**	**IA19^T^**	**A2-NA12**	**A2-NA13**
Genome size (bp)	6,487,705	5,933,382	5,938,884
GC content	59.5	59.7	59.7
Number of contigs	127	84	80
Predicted coding sequences	5,961	5,148	5,172
Subsystems	534	524	531
Number of RNAs	70	68	71
Glycosyl hydrolases (GH)	38	32	34
Polysaccharide lyases (PL)	3	4	4
Carbohydrate esterases (CE)	32	30	32
Carbohydrate-binding modules (CBM)	15	11	10
Auxiliary activities (AA)	22	10	13
Glycosyl transferases (GT)	39	37	38

Draft genome sequences of strains *Pseudomonas bohemica* IA19^T^, A2-NA12 and A2-NA13 were deposited in GenBank under the accession numbers NKHL00000000, PEGA00000000 and PEGB00000000, respectively.

### Genome mining of biosynthetic gene clusters with potential interest in the pharmaceutical industry

Using the SEED viewer, based on the automatic annotation of the bacterial genomes performed with RAST, it was found that each of the genomes of the three bacteria IA19^T^, A2-NA12, and A2-NA13 presented a cluster of genes implicated in the production of the peptide antibiotic colicin V. Also, all strains showed genes implicated in the synthesis, reception and transport of siderophores, molecules implicated not only in the acquisition of iron, but also in microbial inhibition (Becerra et al., 2003). Strains A2-NA12 and A2-NA13 appeared to possess clusters of genes implicated in the synthesis of the siderophores pyoverdine and enterobactin. A gene from a cluster related to achromobactin siderophore was annotated in the draft genome sequence of strain IA19^T^.

The prediction of gene clusters involved in secondary metabolite biosynthesis by antiSMASH version 3.0.5 (Weber et al., 2015) suggested that the genome of the three bacterial isolates contained several potential biosynthetic gene clusters encoding the production of antimicrobial substances, as well as other compounds with potential interest in the pharmaceutical industry, which are listed in Table 6.

**Table 6:** Cluster of genes predicted to encode the synthesis of bioactive compounds in the genome sequences of the strains of this study based on the analysis of genome sequences with AntiSMASH 3.0 program.

**Cluster**	**Type**	**Size (bp)**	**Most similar known cluster (% of similarity)**	MIB – ID^*^
***Pseudomonas*** **IA19**^T^
Cluster 3	–	13,912	Bromophenols and Bromopryrroles biosynthetic genes (20%)	BGC0000891_c1
Cluster 6	–	6,919	Lipopolysaccharide biosynthetic genes (8%)	BGC0000774_c1
Cluster 11	Fatty acid	19,493	Pyoverdine biosynthetic genes (4%)	BGC0000413_c1
Cluster 12	–	19,861	Amphotericin biosynthetic genes (15%)	BGC0000015_c2
Cluster 14	–	23,611	Pyoverdine biosynthetic genes (1%)	BGC0000413_c1
Cluster 22	Saccharide	31,526	Pyoverdine biosynthetic genes (3%)	BGC0000413_c1
Cluster 24	Saccharide	40,812	Pseudopyronine A and Pseudopyronine B biosynthetic genes (62%)	BGC0001285_c1
Cluster 25	Terpene-Siderophore	43,656	Carotenoid biosynthetic genes (85%)	BGC0000642_c1
Cluster 32	Saccharide	50,218	Lipopolysaccharide biosynthetic genes (36%)	BGC0000776_c1
Cluster 37	–	17,662	PM100117 and 100118 biosynthetic genes (10%)	BGC0001359_c1
Cluster 41	–	24,157	2-amino-4-methoxy-trans-3-butenoic acid biosynthetic genes (40%)	BGC0000287_c1
Cluster 42	–	9,467	Caryoynencin biosynthetic genes (16%)	BGC0000892_c1
Cluster 47	–	13,041	Alginate biosynthetic genes (100%)	BGC0000726_c1
***Pseudomonas*** **A2-NA12**
Cluster 1	Saccharide, Fatty acid	34,524	5′-hydroxystreptomycin biosynthetic genes (9%)	BGC0000690_c1
Cluster 2	–	23,963	Polysaccharide B biosynthetic gene (6%)	BGC0001411_c1
Cluster 4	–	28,529	Streptolydigin biosynthetic gene (5%)	BGC0001046_c1
Cluster 14	–	25,995	Lankacidin biosynthetic gene (13%)	BGC0001100_c1
Cluster 16	–	12,110	Alginate biosynthetic genes (100%)	BGC0000726_c1
Cluster 22	–	14,085	9-methylstreptimidone biosynthetic gene (9%)	BGC0000171_c1
Cluster 23	NRPS	52,992	Pyoverdine biosynthetic genes (22%)	BGC0000413_c1
Cluster 26	Saccharide	4,662	Lipopolysaccharide biosynthetic genes (36%)	BGC0000776_c1
Cluster 28	Bacteriocin	10,839	Bacillomycin_biosynthetic_gene_cluster (20%)	BGC0001090_c1
Cluster 34	NRPS	129,458	Pyoverdine biosynthetic gene (18%)	BGC0000413_c1
***Pseudomonas*** **A2-NA13**
Cluster 3	–	10,565	Lipopolysaccharide biosynthetic genes (5%)	BGC0000774_c1
Cluster 5	–	23,657	Polysaccharide B biosynthetic genes (6%)	BGC0001411_c1
Cluster 6	Bacteriocin	10,839	Bacillomycin B biosynthetic genes (20%)	BGC0001090_c1
Cluster 7	NRPS	1,333,658	Pyoverdine biosynthetic gene (21%)	BGC0000413_c1
Cluster 13	–	18,593	9-methylstreptomidone biosynthetic gene (9%)	BGC0000171_c1
Cluster 21	–	17,344	Meilingmycin biosynthetic gene (3%)	BGC0000093_c1
Cluster 27	–	25,977	Lankacidin biosynthetic gene (13%)	BGC0001100_c1
Cluster 28	NRPS	52,992	Pyoverdine biosynthetic gene (21%)	BGC0000413_c1
Cluster 31	Saccharide	46,647	Lipopolysaccharide biosynthetic gene (36%)	BGC0000776_c1
Cluster 38	Fatty acid, Saccharide	32,344	5′-hydroxystreptomycin biosynthetic gene (9%)	BGC0000690_c1
Cluster 40	–	24,316	Streptolydigin biosynthetic gene (5%)	BGC0001046_c1

* *Minimum Information about a Biosynthetic Gene Cluster repository*.

Specifically, the draft genome of isolate IA19^T^ included 49 gene clusters related to the synthesis of secondary metabolites. Out of these, 13 were predicted to be related to already described metabolic pathways involved in antimicrobials production: 2 polyketide synthases (PKSs), 2 non-ribosomal peptide synthetases (NRPS), a terpene-siderophore hybrid, 2 bromophenols (caryoynecins), 5 saccharides and a fatty acid.

In the case of strain A2-NA12, its draft genome contained 37 clusters encoding secondary metabolites, of which 10 are related to described clusters of genes implicated in the synthesis of enzymes related to the production of antimicrobial compounds: 2 NRPS, a PKS, a bacteriocin, 3 saccharides, a NRPS-PKS hybrid, a NRP-PKS-saccharide hybrid and a fatty acid-saccharide hybrid.

Finally, the draft genome of strain A2-NA13 included 40 clusters encoding the synthesis of secondary metabolites, where 10 of them were associated to known metabolic pathways for antimicrobials synthesis. These included 2 PKS, a lipopolysaccharide, a bacteriocin, 2 NRPS, a NRPS-PKS hybrid, a saccharide, a fatty acid-saccharide hybrid and a NRPS-PKS-saccharide hybrid.

## Discussion

Several recent studies have suggested the implication of some bacteria associated with bark beetles in the protection of the bark beetle holobiont. This occurs through the inhibition of antagonists of the beetle itself or the beetle's symbionts, or the detoxification of the bark beetle environment (García-Fraile, 2018). Indeed, all bacterial isolates from this study were able to reduce or inhibit *Aspergillus*, a fungus which has been shown to greatly reduced the number of larvae in the mountain pine beetle (Therrien et al., 2015) and the spruce bark beetle (Cardoza et al., 2006).

In this study, it has been shown that many bacterial isolates from bark beetles, such as *I. acuminatus, C. piceae, P. pityographus*, and *P. bidentatus*, have the potential to produce antimicrobial compounds. Therefore, the genetic potential of some of these isolates to produce antibiotics, as well as other bioactive compounds was further analyzed. Bacteria from the genus *Pseudomonas* are frequently isolated from bark beetles of different species and life cycle stages (Adams et al., 2009, 2013; Morales-Jiménez et al., 2012; Menéndez et al., 2015; Fabryová et al., 2018; García-Fraile, 2018). Members of the genus *Pseudomonas* have been broadly studied for their capability to produce several different secondary metabolites with potential biotechnological applications. In several articles, different strains of *Pseudomonas* are described as biological control agents of plant diseases (Haas and Keel, 2003) and bioactive substances described from *Pseudomonas* strains are diverse, and include quinolines, pyrroles, pseudopeptide pyrrolidinediones, pseudopyronines, siderophores, phthalates, phenazine, phloroglucinol, benzaldehyde, phenanthren, moiramides, andrimid, zafrin, caryoynencin, and bushrin (Pierson et al., 1994; Sarniguet et al., 1995; Schnider et al., 1995; Yamaguchi et al., 1995; Raaijmakers et al., 1997; Isnansetyo and Kamei, 2009; Marinho et al., 2009; Bauer et al., 2015; Ganne et al., 2017). Therefore, these bacteria may be implicated in the protection of the bark beetle by inhibiting microbial antagonists. In this sense, it was observed that bacteria belonging to this genus were among the best microbial inhibitors in our study. Thus, the genomes of those isolates identified as *Pseudomonas* were selected and sequenced, and the exploration of their metabolic capacity to produce bioactive compounds revealed the presence of numerous clusters that are potentially implicated in the synthesis of important bioactive metabolites (Table 6).

A cluster related to the synthesis of 2-amino-4-methoxy-3-butenoic acid, which is a compound also found in *P. aeruginosa* that has been described as being antitumoral (Tisdale, 1980) and to have antiparasitic activity against *Acantamoeba castellani* and antimicrobial activity against *Erwinia amylovora, Bacillus* spp. and *Escherichia coli* (Lee et al., 2010, 2012, 2013), was predicted in strain IA19^T^.

A cluster of genes for the synthesis of PM100117 and PM100118, two bioactive polyhydroxyl macrolide lactones, which have been isolated from the culture broth of the marine-derived *Streptomyces caniferus* (Pérez et al., 2016), was discovered in the genome sequence of strain IA19^T^. It has been shown that these molecules have antitumoral activity as well as slight antifungal activity against *Candida albicans* (Pérez et al., 2016).

A cluster related to the synthesis of caryoynencin, an antibiotic found in liquid cultures of the plant pathogen *Pseudomonas caryophylli* (Yamaguchi et al., 1995) and in a *Burkholderia* sp. isolate from a beetle (Flórez et al., 2017), showing potent antimicrobial activities against Gram-positive and Gram-negative bacteria, as well as antifungal activity, has been predicted in the genome sequence of strain IA19^T^. The spectrum of activity of the described caryoynencin includes: methicillin-resistant *Staphylococcus aureus, Bacillus subtilis, Enterococcus faecalis, Escherichia coli*, “*Salmonella enteritidis*,” *Klebsiella pneumoniae, Serratia marcescens, Proteus vulgaris, Shigella flexneri, Enterobacter cloacae, P. aeruginosa, Candida albicans*, “*Cryptococcus neoformans*,” *Mucor mucedo, Aspergillus fumigatus, Microsporum gypseum, Trichophyton mentagrophytes, Trichophyton interdigitale*, and *Trichophyton rubrum* (Yamaguchi et al., 1995).

In the genome sequences of the three isolates, a cluster related to the synthesis of a lipopolysaccharide compound, also found in *Escherichia coli* and described as a potent inhibitor of HIV-1 replication in T lymphocytes and macrophages (Verani et al., 2002), was predicted.

A cluster for the synthesis of a carotenoid was predicted in the genome sequence of strain IA19^T^. Carotenoids have been reported to have many health benefits, such as prevention of cancer, improvement of visual function and enhancement of immune responses (Sedkova et al., 2005).

A cluster for the synthesis of an alginate, which has been described to improve the efficacy of some antibiotics (Onsoyen et al., 2010), has been predicted in the genome sequence of strains IA19^T^ and A2-NA12.

In the genome sequence of strain IA19^T^, a cluster for the synthesis of pseudopyronines A and B was predicted. Both substances have antibacterial activity based on selective membrane disruption and inhibition of fatty-acid synthase against *Mycobacterium tuberculosis, Bacillus subtilis, Pseudomonas savastanoi*, methicillin-resistant *Staphylococcus aureus, Moraxella catarrhalis*, and vancomycin-resistant Enterococci. This strain also displayed moderate inhibition of other *Firmicutes* (*Listeria welshimeri*) and *Actinobacteria* (*Micrococcus luteus, Arthrobacter crystallopoietes*, and *Corynebacterium xerosis*), as well as anti-leishmanial and algaecide activities (Bauer et al., 2015). Moreover, pseudopyronine B has also shown antitumoral activity (Nishanth Kumar et al., 2016). These compounds have been identified in different species of the genus *Pseudomonas* and in the genus *Alteromonas* (Bauer et al., 2015).

In the genome sequences of the three bacterial isolates, clusters related to the synthesis of pyoverdine, a siderophore and an iron chelating agent produced also be *P. aeruginosa* (Ganne et al., 2017), which can act as antibacterial (Becerra et al., 2003), have been predicted. This molecule has also been described as producer of oxidative stress in leukocytes (Becerra et al., 2003). Moreover, when pyoverdina is conjugated with antibiotics it facilitates them to overcome the bacterial membrane (Kinzel et al., 1998; Kinzel and Budzikiewicz, 1999), which could be used against antibiotic resistance.

A cluster of genes predicted to be encoding the synthesis of the bromopyrrole pentabromopseudilin was found within the genome sequence of strain IA19^T^. Pentabromopseudilin was isolated for the first time from *Alteromonas luteoviolacea* (Laatsch et al., 1995). It is the most active member in a group of more than 20 pyrrole antibiotics that effectively interferes with the macromolecular syntheses in Gram-positive and Gram-negative bacteria, and also has antifungal activity, as well as the activity against the biosynthesis of cholesterol. In addition, it shows pronounced *in vitro* activity against experimental leukemia and melanoma cell lines (Laatsch et al., 1995).

In the genome sequences of isolates A2-NA12 and A2-NA13, a cluster related to the synthesis of 5′-hydroxystreptomycin was predicted. This compound is an aminoglycoside antibiotic that can be biosynthesized by different *Streptomyces* species (Beyer et al., 1998).

The genome sequences of isolates IA19^T^ show a cluster of genes for the biosynthesis of amphotericin, a polyene macrolide produced by *Streptomyces nodosus*, which is a potent antifungal compound and also has activity against some viruses, protozoans and prions (Caffrey et al., 2001).

A cluster of genes related to that encoding the biosynthesis of streptolydigin, a tetramic acid also produced by *Streptomyces lydicus*, has been predicted in the genome sequences of strains A2-NA12 and A2-NA13. This compound is a potent antibiotic which inhibits the bacterial RNA polymerase. It can be also used in acute limphoblastic leukemia or even in acute and chronic myelocytic leukemia (Olano et al., 2009).

The genomes of strains A2-NA12 and A2-NA13 both contain a group of genes that have been related to clusters of genes from *Streptomyces*, which have been described to encode the synthesis of lankacidin, a macrocyclic antibiotic and antitumor against leukemia (Arakawa et al., 2005).

Also, in the genome sequences of strains A2-NA12 and A2-NA13, there are cluster of genes related to other genes implicated in the biosynthesis of 9-methylstreptimidone. This substance is an antifungal (Allen et al., 1976) and antiviral agent which inhibits the growth of polyvirus, vesicular stomatitis virus (VSV) and Newcastle disease virus (NDV) (Saito et al., 1974) and also inhibit NF-κB (nuclear factor-κB) (Ishikawa et al., 2009).

Clusters related to the synthesis of bacillomycin have been predicted in strains A2-NA12 and A2-NA13. This antibiotic, described in *Bacillus subtilis*, possesses antifungal activity against practically all the important dermatophytes and systemic infectious fungi (Landy et al., 1948).

A cluster related to the synthesis of meilingmycin, which possesses potent, broad-spectrum anthelmintic, insecticidal and acaricidal activities and is produced by *Streptomyces nanchangensis* (Zhuang et al., 2006), has been found in the genome sequence of the strain A2-NA13.

As shown above, the selected isolates encoded a large number of clusters involved in the synthesis of secondary metabolites. The strains encoded in their genomes 37 (A2-NA12), 40 (A2-NA13), and 49 (IA19^T^) of these clusters. These values are higher than the average of clusters found in the genomes of other *Pseudomonas* strains isolated from different origins. For example, recently published genomes of *Pseudomonas* strains isolated from soil or rhizosphere encode 9 (Hennessy et al., 2015), 13 (Vida et al., 2017) or 16 (Adam et al., 2015) of these clusters; in the same way, *Pseudomonas* strains isolated from plant tissues present 4 (Wemheuer et al., 2017) or 6 (Maggini et al., 2017) of these clusters. Most of these new genomes have revealed new potentially bioactive compounds in this genus, but importantly, many of the substances predicted in the genome sequences of the strains of this study have never been identified in other *Pseudomonas* strains. Moreover, the low similarity found between some of the described predicted clusters and those of the microorganisms in the AntiSMASH database seem to indicate that several of those substances are potential new chemical compounds, which increases the interest of these bacteria as potential producers of new medical drugs. These results are in accordance with previous findings in the genomes of bacterial symbionts in other insects (Arnam et al., 2018), demonstrating the large variety of cryptic metabolites encoded by these strains, of which only a small quantity have been characterized so far. Therefore, the genomic information and the clusters of genes predicted to be involved in secondary metabolite biosynthesis of the bacterial isolates of this study will require further analysis regarding the structure and function of the bioactive compounds encoded in their genomes.

Our results show that several *Pseudomonas* strains associated with bark beetles possess the genetic potential to produce several antimicrobial substances, as well as other chemical compounds with pharmaceutical interest. Although the bacterial strains identified as *Pseudomonas* were selected and their genome sequences studied, many other bark beetle isolates screened in this study for the capacity to produce antimicrobials seem to have great potential as producers of bioactive compounds. The genome sequencing and mining of other isolates, as well as the production, purification and identification of the predicted bioactive compounds *in silico* are necessary and could have a potential positive impact on the availability of new compounds for the development of drugs.

Regarding the taxonomic study of the strain IA19^T^, the analysis of its 16S rRNA sequence supports its classification within the genus *Pseudomonas*. Nevertheless, the phylogenetic analysis of this gene sequence, as well as the sequences of the housekeeping genes, and those genes of related species within the genus, suggests that IA19^T^ is a new species of the genus *Pseudomonas*. Furthermore, ANI values with the type strains of its closest related species confirm that this strain belongs to a new species. On the other hand, our isolate differs from its closest related species in several phenotypic characters. Upon considering all phenotypic, chemotaxonomic and phylogenetic data of this study, the strain IA19^T^ appears to represent a novel species within the genus *Pseudomonas*, for which the name *Pseudomonas bohemica* sp. nov. is proposed.

### Description of *Pseudomonas bohemica* sp. nov.

*Pseudomonas bohemica* (bo.he'mi.ca. M.L.adj. related to Bohemia, the region in the Czech Republic were the type strain was isolated). Temperature for growth ranges between 4 and 37, growth pH ranges between 6.5 and 8. Optimal growth occurs at 25°C and pH 7–8. Able to grow with up to 2.5% NaCl in TSB. The respiratory ubiquinones are Q9 (97%) and Q8 (3%). C16:0 (30.5%) and in summed feature 3 (20.1%) are the main fatty acids. Oxidase- and catalase-positive. In the API20NE system, aesculin hydrolysis, assimilation of D-glucose, L-arabinose, D-mannose, D-mannitol, potassium gluconate and trisodium citrate are positive whereas reduction of nitrates, glucose fermentation, production of gelatinase, urease, indole arginine dihydrolase and ß-galactosidase and assimilation of adonitol, methyl-xyloside, N-acetyl glucosamine, D-maltose, caprate, adipate, malate and phenylacetate are negative. In API 50CH, it produces acid from D-glucose, glycerol, ribose, L-xylose, L-fructose, D-mannose, L-arabinose, amigdalin, arbutine and salicin.

The type strain, IA19^T^ (=CECT 9403^T^ = LMG 30182^T^), was isolated from a bark beetle from the species *Ips acuminatus* in the Czech Republic. The DNA G+C content of the type strain is 59.5 mol%.

## Author contributions

ZS-S, AJ-G, AD-M, JI performed the experiments. MK made the sampling. RL-M, TV, ZS-S, AJ-G, PG-F performed the bioinformatic analysis of the data. PG-F and RR designed the research project. PG-F, ZS-S, RL-M, RR, EV analyzed the data. PG-F, ZS-S, AJ-G, EV interpreted the results. ZS-S and PG-F contributed to the writing of the manuscript.

### Conflict of interest statement

The authors declare that the research was conducted in the absence of any commercial or financial relationships that could be construed as a potential conflict of interest.

## Supplementary material

Supplementary Figure 1 Neighbor-joining phylogenetic tree based on nearly complete (1,400 bp) 16S rRNA gene sequences of all *Pseudomonas* species closely related to *P. bohemica* IA19^T^ and the species *Acinetobacter baumannii* DSM30007 ^T^, which was included as an outgroup. Bootstrap values (expressed as percentages of 1,000 replications) are shown at the branching points. Scale bar = 2 nucleotides (nt) substitutions per 100 nt. Accession numbers of the sequences are indicated in parentheses.

Supplementary Figure 2 Neighbor-joining phylogenetic tree based on concatenated partial *gyr*B, *rpo*B, *rpo*D and gene sequences of strain *P. bohemica* IA19^T^ and closely related species of the genus *Pseudomonas*. Bootstrap values (expressed as percentages of 1,000 replications) are shown at the branching points. Scale bar = 2 nucleotides (nt) substitutions per 100 nt. Accession numbers of the sequences are indicated in parentheses.
