# Supplementary figures and images for: Discovery of Phloeophagus Beetles as a Source of Pseudomonas Strains That Produce Potentially New Bioactive Substances and Description of Pseudomonas bohemica sp. nov
Source: Front Microbiol. 2018 May 8;9:913. doi: 10.3389/fmicb.2018.00913 (PMC5953339; doi:10.3389/fmicb.2018.00913)

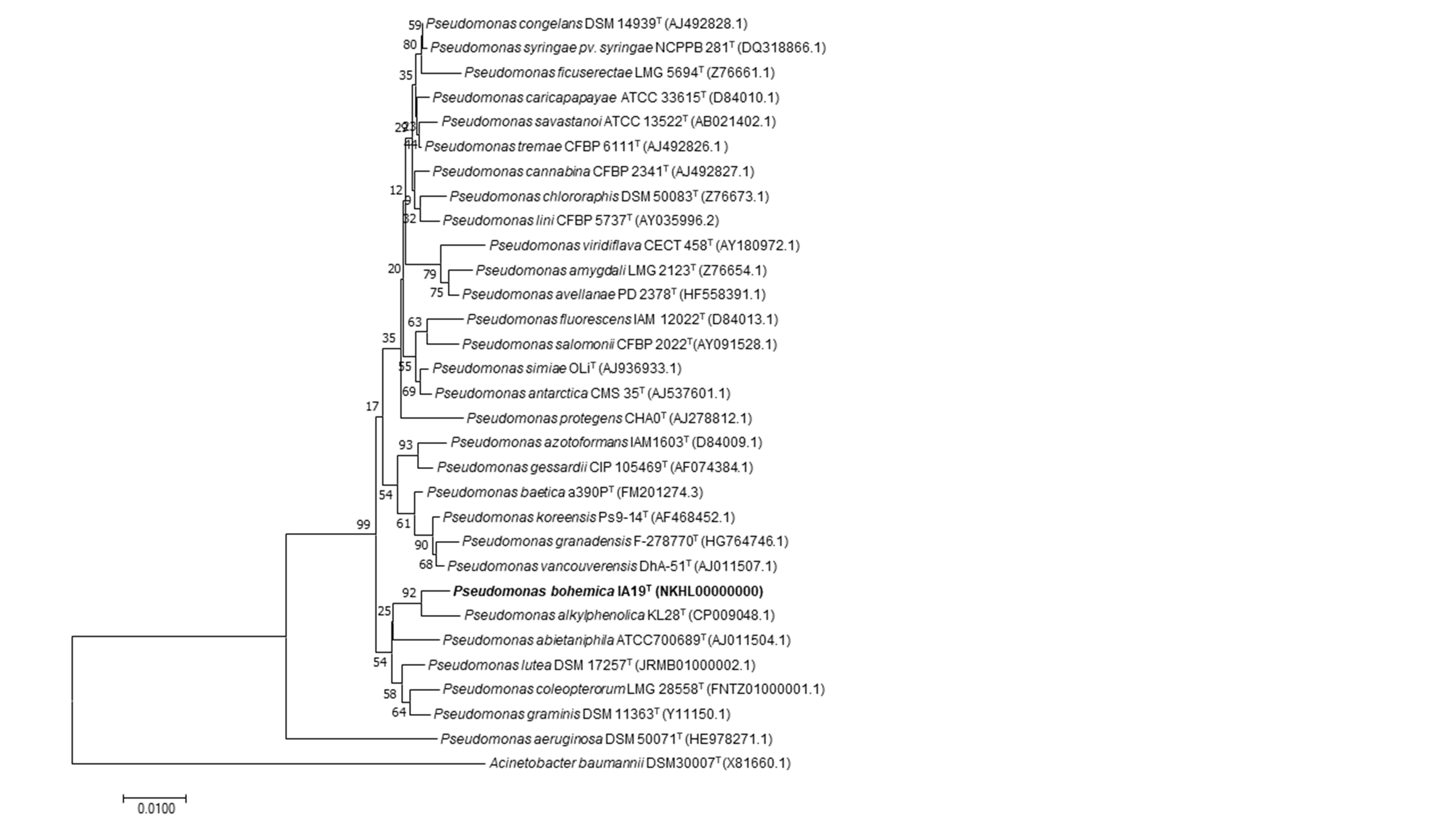

Supplement: Supplementary Figure 1 — Neighbor-joining phylogenetic tree based on nearly complete (1,400 bp) 16S rRNA gene sequences of all Pseudomonas species closely related to P. bohemica IA19T and the species Acinetobacter baumannii DSM30007 T, which was included as an outgroup. Bootstrap values (expressed as percentages of 1,000 replications) are shown at the branching points. Scale bar = 2 nucleotides (nt) substitutions per 100 nt. Accession numbers of the sequences are indicated in parentheses. [file Image_1.TIF]

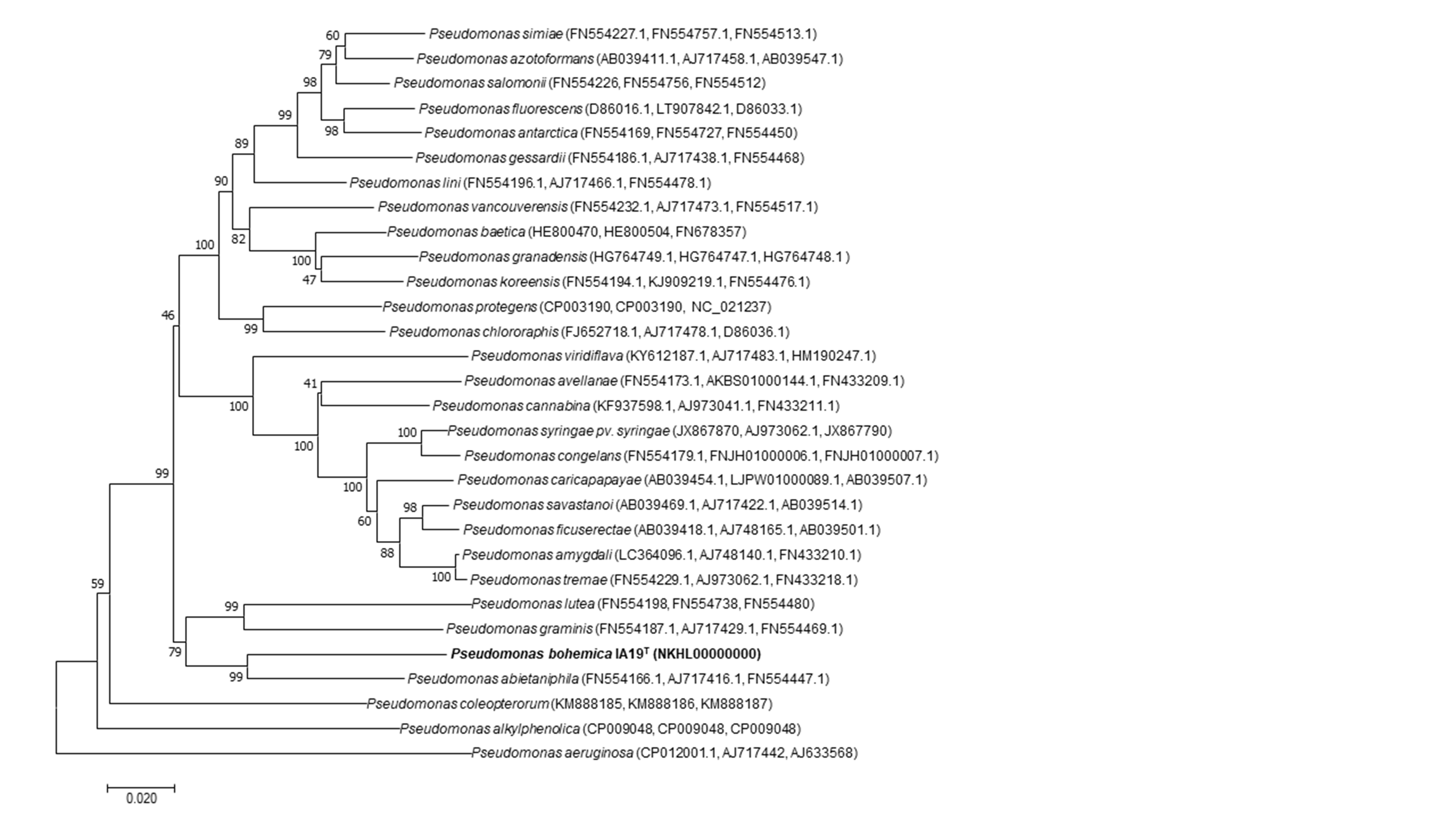

Supplement: Supplementary Figure 2 — Neighbor-joining phylogenetic tree based on concatenated partial gyrB, rpoB, rpoD and gene sequences of strain P. bohemica IA19T and closely related species of the genus Pseudomonas. Bootstrap values (expressed as percentages of 1,000 replications) are shown at the branching points. Scale bar = 2 nucleotides (nt) substitutions per 100 nt. Accession numbers of the sequences are indicated in parentheses. [file Image_2.TIF]
